# Supplementary material for: Potent neutralization of clinical isolates of SARS-CoV-2 D614 and G614 variants by a monomeric, sub-nanomolar affinity nanobody
Source: Sci Rep. 2021 Feb 8;11:3318. doi: 10.1038/s41598-021-82833-w (PMC7870875; doi:10.1038/s41598-021-82833-w)
Supplement: Supplementary file 1 — Supplementary information. [file 41598_2021_82833_MOESM1_ESM.pdf]

# Potent neutralization of clinical isolates of SARS-CoV-2 D614 and G614 variants by a monomeric, sub-nanomolar affinity Nanobody.

Guillermo Valenzuela Nieto[1], Ronald Jara[1 & 2], Daniel Watterson[3, 4 & 5], Naphak Modhiran[3 & 4], Alberto A Amarilla[3], Johanna Himelreichs[1], Alexander A. Khromykh[3 & 5], Constanza Salinas-Rebolledo[1], Teresa Pinto[1], Yorka Cheuquemilla[1 & 8], Yago Margolles[6], Natalia López González del Rey[7], Zaray Miranda-Chacon[1], Alexei Cuevas[1], Anne Berking[8], Camila Deride[1 & 11], Sebastián González-Moraga[1], Héctor Mancilla[1], Daniel Maturana[9], Andreas Langer[9], Juan Pablo Toledo[1], Ananda Müller[10 & 11], Benjamín Uberti[11], Paola Krall[1 & 12], Pamela Ehrenfeld [13 & 17], Javier Blesa[7], Pedro Chana-Cuevas[14], German Rehren[15], David Schwefel[16], Luis Ángel Fernandez[6] & Alejandro Rojas-Fernandez[1, 8,17 & 18]

1 Institute of Medicine, Faculty of Medicine, Universidad Austral de Chile, Valdivia, Chile.

2 Institute of Biochemistry and Microbiology, Faculty of Sciences, Universidad Austral de Chile, Valdivia, Chile.

3 School of Chemistry and Molecular Bioscience, The University of Queensland, Brisbane, Australia

4 The Australian Institute for Biotechnology and Nanotechnology, The University of Queensland, Brisbane, Australia

5 Australian Infectious Diseases Research Centre, The University of Queensland, Brisbane, Australia

6 Department of Microbial Biotechnology, National Biotechnology Center, Superior Council of Scientific Research, Madrid, Spain.

7 HM CINAC, Hospital Universitario HM Puerta del Sur, Mostoles 28938, Madrid, Spain

8 Berking Biotechnology, Valdivia, Chile.

9 NanoTemper Technologies GmbH, Floessergasse 4, 81369 Munich, Germany.

10 Ross University School of Veterinary Medicine, Saint Kitts and Nevis, West Indies.

11 Institute of Veterinary Clinical Sciences, Faculty of Veterinary Sciences, Universidad Austral de Chile, Valdivia, Chile.

12 Department of Pediatrics and Children's Surgery Oriente, Universidad de Chile.

13 Institute of Anatomy, Histology, and Pathology, Faculty of Medicine, Universidad Austral de Chile.

14 CETRAM & Faculty of Medical Science Universidad de Santiago de Chile, Chile.

15 Technology Transfer and Licensing office, Universidad Austral de Chile, Valdivia, Chile.

16 Charité – Universitätsmedizin Berlin, corporate member of Freie Universität Berlin, Humboldt-Universität zu Berlin, and Berlin Institute of Health, Berlin, Germany

17 Center for Interdisciplinary Studies on the Nervous System, CISNE, Universidad Austral de Chile, Valdivia, Chile.

18 Institute of Philosophy and Complexity Sciences, Santiago, Chile.

* Correspondence should be addressed to alejandro.rojas@uach.cl

## Supplemental Figure Legends

**Supplemental Figure-1.** Evaluation of the alpaca´s immune response by ELISA. Sera before and after the second immunization (3 weeks) have been diluted as indicated in the figure and total IgG1, IgG2 & IgG3 have been evaluated to recognize full length Spike before and after immunization, n=4, error bars indicate standard deviation.

**Supplemental Figure-2. Controls and expression**. **A)** Immunofluorescence assays of Spike-GFP transfected HeLa cells using purified recombinant W25UACh nanobody as primary antibody. **B)** Immunofluorescence assays of Nucleoprotein- GFP transfected HeLa cells using purified recombinant W25UACh nanobody as primary antibody**.**

**Supplemental Figure-3. Original scan and supplemental neutralization table. A)** Original figure 2a. **B)** original Figure 3a **C)** Zoom image of W25 inflection of Figure 2b, **D)** Summary table of neutralizing activity of W25 compared to previously reported Nbs

## Nanobodies Sequences

### Nucleotide sequences W23 Nanobody

ATGGCTCAGGTGCAGCTGGTGGAGTCTGGGGGAGGCTTGGTGCAGCCTGGGGAGTCTCTGAGACTCTC
CTGTGCAGCCTCTGGAAACATCTTCGGAATCGCTGCCGTGCACTGGTTCCGCAAGGCTCCAGGGAAGGA
GCGCGAGTTTACTGCAGGTTTTGGTAGTGATGGTAGCACAAACTATGCAAACTCCGTGAAGGGCCGATT
CACCATCTCCAGAGACAATGCCAAGAACACGACATATCTGCAAATGAACAGCCTGAAACCTGAGGACAC
GGCCGTCTATTATTGTCATGCGCTAATCAAGAATGAACTTGGATTCCTTGATTACTGGGGCCCGGGGACC
CAGGTCACCGTCTCCTCA

### Amino acid sequences W23 Nanobody

MAQVQLVESGGGLVQPGESLRLSCAASGNIFGIAAVHWFRKAPGKEREFTAGFGSDGSTNYANSVKGRFTIS
RDNAKNTTYLQMNSLKPEDTAVYYCHALIKNELGFLDYWGPGTQVTVSS

### Nucleotide sequences W25 Nanobody

ATGGCTCAGGTGCAGCTGGTGGAGTCTGGGGGAGGCTTGGTGCAGCCTGGGGAGTCTCTGAGACTCTC
CTGTGCAGCCTCTGGAAGTATCTTCGGAATCTATGCCGTGCACTGGTTCCGCATGGCTCCAGGGAAGGA
GCGCGAGTTTACTGCAGGTTTTGGAAGTCATGGTAGCACAAATTATGCAGCTTCCGTGAAGGGACGATT
CACCATGTCCAGAGACAATGCCAAGAACACGACGTATCTGCAAATGAACAGCCTGAAACCTGCGGACAC
GGCCGTCTATTACTGTCATGCGCTAATAAAGAATGAACTTGGGTTCCTTGACTACTGGGGCCCGGGGAC
CCAGGTCACCGTCTCCTCA

### Amino acid sequences W25 Nanobody

MAQVQLVESGGGLVQPGESLRLSCAASGSIFGIYAVHWFRMAPGKEREFTAGFGSHGSTNYAASVKGRFT
MSRDNAKNTTYLQMNSLKPADTAVYYCHALIKNELGFLDYWGPGTQVTVSS
